# Characterizing the neurological phenotype of the hyperinsulinism hyperammonemia syndrome

**DOI:** 10.1186/s13023-022-02398-3

**Published:** 2022-06-25

**Authors:** Elizabeth Rosenfeld, Ravi Prakash Reddy Nanga, Alfredo Lucas, Andrew Y. Revell, Allison Thomas, Nina H. Thomas, David R. Roalf, Russel T. Shinohara, Ravinder Reddy, Kathryn A. Davis, Diva D. De León

**Affiliations:** 1 Division of Endocrinology and Diabetes, Children’s Hospital of Philadelphia, 3500 Civic Center Boulevard, Philadelphia, PA 19140 USA; 2 Congenital Hyperinsulinism Center, Children’s Hospital of Philadelphia, Philadelphia, PA USA; 3 Department of Pediatrics, Perelman School of Medicine, University of Pennsylvania, Philadelphia, PA USA; 4 Center for Advanced Metabolic Imaging in Precision Medicine, Hospital of the University of Pennsylvania, Philadelphia, PA USA; 5 Penn Center for Neuroengineering and Therapeutics, University of Pennsylvania, Philadelphia, PA USA; 6 Behavioral Neuroscience Core, Center for Human Phenomic Science, Children’s Hospital of Philadelphia, Philadelphia, PA USA; 7 Department of Child and Adolescent Psychiatry and Behavioral Sciences, Children’s Hospital of Philadelphia, Philadelphia, PA USA; 8 Department of Psychiatry, University of Pennsylvania Perelman School of Medicine, Philadelphia, PA USA; 9 Penn Statistics in Imaging and Visualization Center, Department of Biostatistics, Epidemiology, and Informatics, University of Pennsylvania Perelman School of Medicine, Philadelphia, PA USA; 10 Center for Biomedical Image Computing and Analytics, Department of Radiology, University of Pennsylvania Perelman School of Medicine, Philadelphia, PA USA; 11 Department of Neurology, University of Pennsylvania Perelman School of Medicine, Philadelphia, PA USA

**Keywords:** GLUD1, Hypoglycemia, Epilepsy, Glutamate, GluCEST

## Abstract

**Background:**

Hyperinsulinism hyperammonemia (HI/HA) syndrome is caused by activating mutations in *GLUD1*, encoding glutamate dehydrogenase (GDH). Atypical absence seizures and neuropsychological disorders occur at high rates in this form of hyperinsulinism. Dysregulated central nervous system (CNS) glutamate balance, due to GDH overactivity in the brain, has been hypothesized to play a role. This study aimed to describe the neurologic phenotype in HI/HA syndrome and investigate CNS glutamate levels using glutamate weighted chemical exchange saturation transfer magnetic resonance imaging (GluCEST MRI). In this cross-sectional study, 12 subjects with HI/HA syndrome had plasma ammonia measurement, self- or parent-completed neurocognitive assessments, electroencephalogram (EEG), and GluCEST MRI at 7 T performed. GluCEST MRI measures were compared to a historic reference population of 10 healthy adults.

**Results:**

Subjects were five males and seven females with median age of 25.5 years. Seventy-five percent of subjects reported a history of neurodevelopmental problems and 42% had neurocognitive assessment scores outside the normal range. Fifty percent had interictal EEG findings of generalized, irregular spike and wave discharges. Higher variability in hippocampal GluCEST asymmetry (*p* = 0.002), and in peak hippocampal GluCEST values (*p* = 0.008), was observed in HI/HA subjects (n = 9 with interpretable MRI) compared to the healthy reference population (n = 10).

**Conclusions:**

The high prevalence of abnormal neurocognitive assessment scores and interictal EEG findings observed highlights the importance of longitudinal neuropsychological assessment for individuals with HI/HA syndrome. Our findings demonstrate the potential application of GluCEST to investigate persistent knowledge gaps in the mechanisms underlying the unique neurophenotype of this disorder.

**Supplementary Information:**

The online version contains supplementary material available at 10.1186/s13023-022-02398-3.

## Background

Hyperinsulinism hyperammonemia (HI/HA) syndrome is the second most common genetic form of congenital hyperinsulinism (HI) [1]. HI/HA is caused by dominant activating mutations in the *GLUD1* gene, encoding glutamate dehydrogenase (GDH) [2]. GDH is a mitochondrial enzyme highly expressed in pancreas, liver, kidney, and brain, that catalyzes the reversible conversion of glutamate to alpha-ketoglutarate and ammonia [3, 4]. As with other forms of HI, hyperinsulinemic hypoglycemia is a cardinal feature—in HI/HA, hypoglycemia is triggered by both fasting and protein intake. HI/HA syndrome is additionally characterized by hyperammonemia, and distinctive neurologic manifestations.

Epilepsy in HI/HA is common and is characterized by atypical absence seizures associated with high-amplitude irregular, generalized, spike and wave discharges on electroencephalogram (EEG) [5, 6]. These seizures occur in the setting of euglycemia and are distinct from the focal-onset seizures that may occur following hypoglycemic brain injury [6]. Developmental delays, learning disorders, and attention deficit/hyperactivity disorder (ADHD) are also more prevalent in HI/HA syndrome than in other genetic forms of HI [5, 7, 8]. The pathophysiology of these neurologic manifestations is insufficiently explained by hypoglycemia alone and has not been elucidated. Altered central nervous system (CNS) glutamate concentrations due to GDH overactivity have been hypothesized to play a role, but investigations to confirm this hypothesis have been limited.

Recent advances in magnetic resonance imaging (MRI) techniques have allowed for sensitive estimation of in vivo CNS glutamate concentrations using glutamate weighted chemical exchange saturation transfer (GluCEST). In this technique, amine protons of glutamate are selectively saturated using narrow bandwidth radiofrequency irradiation. These saturated protons exchange freely with water protons, thereby attenuating the water signal, permitting indirect detection of the glutamate concentration [9]. GluCEST imaging has been shown to have higher sensitivity and better spatial resolution than traditional methods, such as magnetic resonance spectroscopy (MRS), for measuring glutamate in humans, including those with neuropathology [9–12]. This study aimed to utilize GluCEST imaging, in conjunction with EEG and neurocognitive assessments, to better characterize the biochemical and clinical neurologic phenotype of HI/HA syndrome.

## Methods

In this cross-sectional study conducted at Children’s Hospital of Philadelphia and the University of Pennsylvania, 12 subjects with biochemically and/or genetically confirmed HI/HA syndrome underwent plasma ammonia measurement, neurocognitive assessments, EEG, and GluCEST MRI at 7 Tesla (7 T). A historic reference population of 10 healthy adults that had undergone GluCEST imaging using the same scanner, MRI acquisition protocol, and image processing pipelines [10] were utilized for GluCEST MRI comparison.

Exclusion criteria were age < 12 years, weight < 30 kg (7 T MRI is FDA approved for individuals weighing > 30 kg), contraindications to MRI scanning (e.g., metallic implant, claustrophobia), investigational drug use in 30 days prior to participation, evidence of active infection or severe organ dysfunction, pregnancy, and limited English proficiency. All subjects and their parent or legal guardian gave their written informed consent and assent, as appropriate, to participate. The study was approved by the Children’s Hospital of Philadelphia Institutional Review Board.

Clinical data were gathered through interview and medical record review. Samples for plasma ammonia measurement were obtained by venipuncture without the use of a tourniquet, placed on ice, and directly transported to the clinical laboratory.

### Neurocognitive measures

Neurocognitive outcomes were assessed through the following self- or parent-administered instruments: the Adaptive Behavior Assessment System, Third Edition (ABAS-3), the Achenbach System of Empirically Based Assessment (ASEBA) Childhood Behavior Checklist (CBCL) for subjects < 18 years old or Adult Self Report (ASR) for subjects ≥ 18 years old, and the Behavior Rating Inventory of Executive Function (BRIEF), Second Edition (BRIEF-2) for subjects < 18 years old or BRIEF-Adult Version (BRIEF-A) for subjects ≥ 18 years old. Forms were completed by a parent for subjects < 18 years of age and were self-completed by subjects ≥ 18 years of age.

The ABAS-3 assesses adaptive skills across the lifespan (birth-89 years). The general adaptive composite (GAC) is the main outcome score of the ABAS-3 and has a mean of 100 and standard deviation (SD) of 15 [13]. The ASEBA CBCL (ages 6–18) and ASR (ages 18–59) assess behavioral, emotional, and social functioning. The ASEBA composite outcome is the total problems (TP) score, which has a mean of 50 and SD of 10 [14]. These measures also include a Diagnostic and Statistical Manual of Mental Disorders, 5th edition-oriented ADHD subscale. The BRIEF-2 and BRIEF-A assess executive function. The main outcome measure is the Global Executive Composite (GEC), which has a mean of 50 and SD of 10 [15, 16]. For the ABAS-3 lower scores indicate worse outcomes, whereas higher scores indicate worse outcomes for the ASEBA and BRIEF measures. Scores on the neurocognitive assessments were considered abnormal if they were > 1 SD below the mean for GAC score (ABAS-3) or > 1 SD above the mean for TP score (ASEBA) or GEC score (BRIEF). This threshold was chosen to permit sensitivity for detecting subtler cognitive effects of potential clinical significance corresponding to below-average scores on the assessment tools utilized. The proportion of subjects with scores > 2 SD below the mean for GAC score, and > 2 SD above the mean for TP score or GEC score, which are considered indicative of clinical concern, was also reported.

### EEG

EEG was acquired on a Natus Neuroworks system using the international 10–20 system for scalp electrode placement. Data were recorded using a sampling frequency of 256 Hz in a referential montage at 30 mm/second speed. Hyperventilation and 15 Hz photic stimulations were performed. EEG interpretation was performed by an epileptologist blinded to GluCEST findings (KAD). Remontaging was performed as clinically indicated for optimal interpretation.

### MRI scans and image analysis

**Fig. 1:**
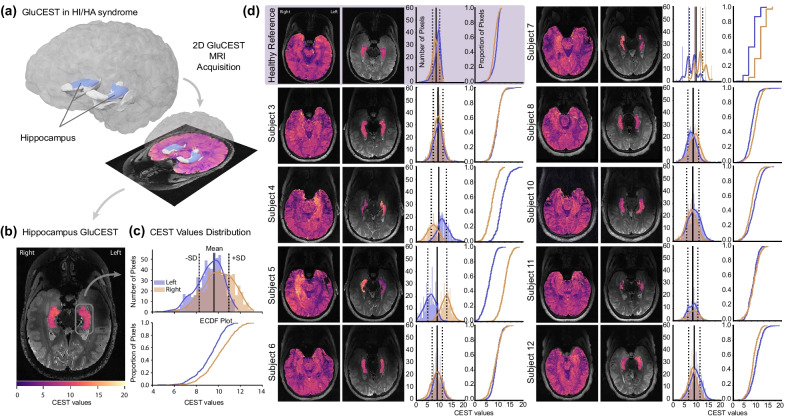
Overview of GluCEST acquisition and analysis. **a** GluCEST is measured on an axial slice at the level of the hippocampus using the same MRI acquisition protocol in nine HI/HA subjects and a reference population of ten healthy adults. Measurements are limited to a single slice (i.e., a single layer of voxels, or equivalently, pixels in 2D). The hippocampus was segmented using the ASHS pipeline in ITK-SNAP. A 3D render of the hippocampus of an example healthy reference adult (C006, see Additional file 1) is shown along with the 2D GluCEST slice of the same individual (note, the 3D render is used for visualization purposes only). **b** The GluCEST values within the hippocampus are overlaid on the subject’s T2 image from panel **a**. **c** The distribution of GluCEST values (top, histogram; bottom, ECDF plot) in each hippocampus is shown corresponding to the same subject of panels **a** and **b**. The solid vertical line represents the mean of all pixel values in the hippocampus, and the dashed vertical lines represent ± 1 SD. **d** Heatmap of GluCEST values are shown for all HI/HA subjects with interpretable MRI and a representative healthy reference subject (C010, see Additional file 1). GluCEST values for both the entire axial slice and within only the hippocampus are shown. Similar to the representative plots of panel **c**, the distribution of hippocampal GluCEST values is also shown. ASHS, Automatic Segmentation of Hippocampal Subfields; CEST, chemical exchange saturation transfer; ECDF, empirical cumulative density function; GluCEST, glutamate chemical exchange saturation transfer; HI/HA hyperinsulinism hyperammonemia syndrome; SD, standard deviation

MRI brain was acquired on each subject using a 7 T Siemens scanner with a single channel transmit and 32-channel receive phased-array head coil. Two-dimensional (2D) GluCEST imaging parameters were as follows: slice thickness = 5 mm, in-plane resolution = 0.8 × 0.8 mm^2^, gradient recalled echo readout TR/TE = 6.2/2.4 ms, number of averages = 2, shot TR = 8000 ms, shots per slice = 2, with an 800-ms-long saturation pulse train consisting of a series of 100-ms pulses at a B_1rms_ of 3.06 µT. Raw CEST images were acquired by varying saturation offset frequencies from ± 1.8 to ± 4.2 ppm (relative to water resonance set as 0 ppm) with a step size of ± 0.3 ppm. In addition, B_0_ and B_1_ maps of the same slices were acquired and used to correct B_0_ and B_1_ inhomogeneities in the GluCEST maps, as described previously [10]. T2-weighted MRI using the variable flip angle turbo spin echo sequence (208 coronal slices, TR/TE = 3000/386 ms, 0.4 × 0.4 × 1.0 mm^3^ resolution, iPAT = 2) was obtained and was used for hippocampal segmentation using the Automatic Segmentation of Hippocampal Subfields (ASHS) pipeline in ITK-SNAP [17]. The B_0_ and B_1_-corrected GluCEST contrast map was then averaged within each hippocampus as shown in Fig. 1a–c. GluCEST asymmetry index (AI) was calculated as the absolute value of the difference between left and right mean hippocampal GluCEST contrast divided by their sum (|left − right|/[left + right] × 100). Peak hippocampal GluCEST was determined as the greater of the right mean hippocampal GluCEST value or left mean hippocampal GluCEST value for each subject. Image processing and analyses were performed with in-house written programs in MATLAB (MathWorks, version 9.7, R2019b) and Python (version 3.6).

### Statistical analysis

One-sample, one-sided z-tests of proportions were used to compare the observed proportion of HI/HA subjects with neurocognitive assessment scores > 1 and > 2 SD out of range with the expected proportions in a normal distribution. Mann–Whitney U tests were used to assess differences in median GluCEST values, and Levene’s test was used to compare variances, between HI/HA subjects and the healthy reference population. An alpha of 0.05 was considered statistically significant. Corrections for multiple comparisons were not conducted due to the exploratory nature of the study*.*

## Results

Demographic and clinical history data are summarized in Table 1. Enrolled subjects consisted of five males and seven females from nine families. Median age at the time of study participation was 25.5 years (range: 13–56 years). Age at HI/HA diagnosis varied considerably, ranging from 1 month to 34 years, with median age of 12 months. In three related subjects (8, 9, and 12), diagnosis was established following the genetic diagnosis of HI/HA syndrome in the infant sibling of subject 12.

**Table 1:** Clinical characteristics of HI/HA subjects

Subject*	Age (y)	Sex	*GLUD-1* mutation^‡^	Treatment	Self-reported neurodevelopmental history
1	32	F	Ser445Leu^§^	Pancreatectomy, age 2y10mo	Normal neurodevelopment
2	16	M	His262Tyr	DZ 9.5 mg/kg/day	ADHD
3*	27	F	Ser448Pro	DZ 2.5 mg/kg/day	Language delay; learning/processing disorder; memory issues
4*	24	F	Ser448Pro	DZ 1 mg/kg/day	Learning/processing disorder
5*	56	M	Ser448Pro	None	Memory issues
6	18	M	Gly446Val	DZ 4 mg/kg/day	Normal neurodevelopment
7	24	F	Arg221Cys^§^	DZ 1 mg/kg/day	Gross motor and language delay; learning disorder
8^†^	13	F	Ala447Thr	DZ 5 mg/kg/day	Learning disorder; memory issues
9^†^	38	M	Ala447Thr	None	Developmental delay
10	28	M	^||^	Pancreatectomy, age 2–3y	Language delay; ADHD; memory issues
11	28	F	^||^	DZ 5 mg/kg/day	Language delay; learning disorder; ADHD
12^†^	13	F	Ala447Thr	DZ 7 mg/kg/day	Learning disorder

*ADHD* attention deficit hyperactivity disorder, *DZ* diazoxide, *F* female, *HI/HA* hyperinsulinism hyperammonemia syndrome, *M* male, *mo* month, *y* year

^*^Subject 5 is the father of subjects 3 and 4

^†^Subject 9 is the father of subject 8 and paternal uncle of subject 12

^‡^*GLUD-1* sequence information is based on GenBank reference sequence NM_005271.3

^§^Mosaic

^||^Genetic testing not performed

Sixty-seven percent of subjects were on diazoxide treatment at the time of participation with mean diazoxide dose of 4.5 ± 2.8 mg/kg/day. Two subjects (1 and 10), diagnosed before identification of *GLUD1* mutation underwent pancreatectomy between 2–3 years of age. Of these, one subject (10) subsequently developed insulin-dependent diabetes at 13 years of age. Of the six subjects (2–4, 7, and 10–11) that endorsed monitoring plasma glucose in the two weeks prior to participation, three had recorded plasma glucose < 3.9 mmol/L (< 70 mg/dL, range: 1–6 events), and one disclosed a single episode of plasma glucose < 3 mmol/L (< 54 mg/dL).

Mean plasma ammonia concentration was 69.0 ± 38.3 µmol/L (normal range: 9.0–33.0 µmol/L). Plasma ammonia was elevated above the normal range in all but two subjects (1 and 7), both of whom had mosaic expression of the *GLUD1* mutations.

### Neurocognitive outcomes

Neurodevelopmental problems were parent or self-reported in 75% of the subjects (Table 1). Learning problems, described as requiring extra support in school, were most common and were reported in 50%. Forty-two percent reported history of delayed developmental milestones, 25% reported history of ADHD, and 25% reported memory problems.

Overall, 42% of the subjects had an abnormal composite score on any of the neurocognitive assessments utilized. On the ABAS-3 measures of adaptive function, the mean GAC score was 98.5 ± 14.6. The proportion of subjects scoring > 1 SD below the mean did not differ significantly from the general population (16.7% vs. 15.8%, *p* = 0.467). No subjects scored > 2 SD below the mean on this measure.

The mean GEC score on the BRIEF measures of executive function was 51.4 ± 15.7. The proportion of subjects scoring > 2 SD above the mean was significantly greater than in the general population (16.7% vs. 2.2%; *p* < 0.001), whereas the proportion scoring > 1 SD above the mean did not significantly differ from the general population (25.0% vs. 15.8%, *p* = 0.191).

On the ASEBA measures assessing behavioral, emotional, and social function, the mean TP score was 48.3 ± 16.4. The proportion of subjects scoring > 1 SD above the mean on these measures did not significantly differ from the general population (16.7% vs. 15.8%, *p* = 0.467), nor did the proportion of subjects scoring > 2 SD above the mean (8.3% vs. 2.2%, *p* = 0.075).

Twenty-five percent of subjects had abnormal scores on the ASEBA ADHD subscale. Two-thirds of those with abnormal ADHD subscale scores self-reported a history of ADHD. In contrast, a relationship between abnormal composite scores on the neurocognitive measures and self-reported neurocognitive problems was not observed; 60% of those with self-reported developmental delays and 83% of those with self-reported learning problems had normal neurocognitive assessment scores.

### Epilepsy outcomes

All subjects had a reported history of seizure onset in infancy or early childhood. Median age of seizure onset was 8.5 months (range: 2 weeks–2 years). Three subjects (2, 4, and 7) reported seizures in the setting of documented euglycemia. For subject 4, this was a single febrile seizure following vaccination. The remainder reported that glycemic status during seizures was typically unknown; hypoglycemia was often presumed given prior history and/or recognized HI/HA diagnosis. Forty-two percent reported prior use of antiepileptic medication, however only one subject (2) remained on treatment (divalproex sodium) for management of absence epilepsy. No other subjects had been diagnosed with absence seizures, although three (8, 9, and 12) reported history of recurrent staring spells. One subject (11) reported experiencing a hypoglycemic seizure in the 12 months prior to participation. The remainder of the untreated subjects reported spontaneous resolution of seizures with age.

**Fig. 2:**
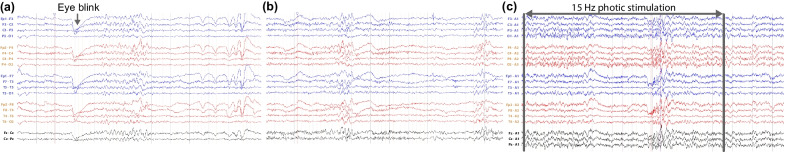
Representative EEG tracings. **a** EEG data from subject 11 demonstrating generalized irregular spike-and-wave discharges at ~ 5 Hz associated with eye blink (arrow) and **b** without eye blink. **c** EEG data from subject 8 displays photosensitive generalized and irregular spike-and-wave discharges. Double-headed, horizontal arrow and vertical lines denote duration of 15 Hz photic stimulation. Normal background EEG activity is observed in both subjects. EEG, electroencephalogram

Fifty-eight percent of the subjects had an abnormality detected on EEG. Interictal abnormalities included generalized irregular spike and wave discharges at 3–6 Hz in six subjects (1, 3, 4, 8, 9, 11), as illustrated in Fig. 2. These findings were additionally associated with eye blinks, rolling, or staring in three subjects (3, 4, 11), bifrontal sharp waves in one (2), and occurred following photic stimulation in two (8, 11). One subject (2) had mild diffuse background slowing with maximal posterior dominant rhythm of 8 Hz. The remainder had normal background rhythm. None had electrographic seizures.

### GluCEST MRI

Interpretable GluCEST MRI results were available for nine subjects. Data for three subjects were not useable due to intolerance of the MRI scan in one subject (9) and motion artifact in two subjects (1, and 2). Median age (24 years [IQR: 18, 28 years] vs. 28 years [IQR: 24, 37 years], *p* = 0.190, Mann–Whitney U test) and sex distribution (67% vs. 70% female, *p* = 0.876, χ^2^ test) were similar between the HI/HA subjects with interpretable scans and the healthy reference population.

**Fig. 3:**
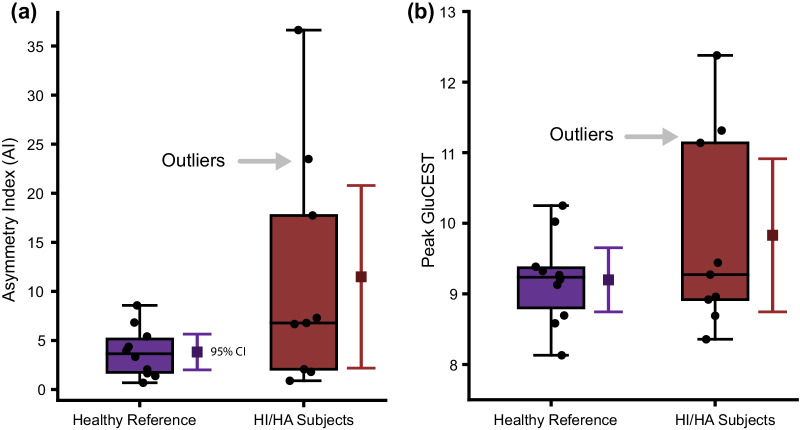
**a** Hippocampal asymmetry index in HI/HA subjects compared to the healthy reference population. *p* = 0.142, Mann–Whitney U test. **b** Peak hippocampal GluCEST in HI/HA subjects compared to the healthy reference population. *p* = 0.514, Mann–Whitney U test. Three subjects (4, 5, and 7) had outlier values (≥ 3 SD) for hippocampal AI using the healthy reference subjects as the reference population distribution. The same three subjects had outlier values for peak hippocampal GluCEST. Asymmetry index calculated as the absolute value of the difference between left and right mean hippocampal GluCEST % divided by their sum (|left − right|/[left + right] × 100). Peak GluCEST determined as the highest mean GluCEST value between the left or right hippocampus of each subject. In the box and whisker plots, horizontal lines represent medians, box ends represent the 25th and 75th percentiles, and whiskers extend to the minimum and maximums. The mean of each group and 95% CI error bars are displayed to the right of each box and whisker plot. *AI* asymmetry index, *CI* confidence interval, *HI/HA* hyperinsulinism hyperammonemia syndrome, *GluCEST* glutamate chemical exchange saturation transfer, *SD* standard deviation

Qualitatively, asymmetric hippocampal GluCEST signal was observed in a subset of HI/HA subjects in contrast to the reference population in whom GluCEST was symmetrical (shown in Fig. 1d). While median hippocampal GluCEST AI did not differ between HI/HA subjects and the healthy reference population (6.78% [IQR: 2.06, 17.74] vs. 3.65% [IQR: 1.66, 5.40], *p* = 0.142, Mann–Whitney U test, shown in Fig. 3a), a statistically significant difference in group variances was observed (*p* = 0.002, Levene’s test). Peak hippocampal GluCEST was calculated and compared between groups to further explore the qualitatively observed asymmetry. Median peak hippocampal GluCEST did not differ between HI/HA subjects and the healthy reference population (9.27% [IQR: 8.92, 11.14] vs. 9.24% [IQR: 8.69, 9.38], *p* = 0.514, Mann–Whitney U test, shown in Fig. 3b). A statistically significant difference in peak hippocampal GluCEST variance between HI/HA subjects and the healthy reference population was observed (*p* = 0.008, Levene’s test).

Three subjects (4, 5, and 7) had hippocampal GluCEST asymmetry indices and peak GluCEST values more than three standard deviations above the mean for the healthy reference population (outliers, shown in Fig. 3). Outliers may be due to technical factors or biological factors. Visual inspection of GluCEST MRI data excluded the former of these, and subsequently, clinical factors were explored. Among these subjects, one had abnormal EEG findings. All self-reported a history of abnormal neurodevelopment, but none had abnormal neurocognitive assessment scores. Mean plasma ammonia was numerically lower in these subjects, compared to the remaining HI/HA subjects with interpretable MRI; however, this difference was not statistically significant (38.3 ± 11.6 µmol/L vs. 79.2 ± 12.7 µmol/L, *p* = 0.112, two-sided t-test).

## Discussion

We report neurophenotype characteristics of 12 patients with HI/HA syndrome, including estimations of CNS glutamate measured by GluCEST MRI. In keeping with recognized features of the HI/HA syndrome, we found a high prevalence of abnormal neurodevelopment. While 75% self-reported a history of neurodevelopmental problems, the prevalence of abnormal neurodevelopment as measured by the neurocognitive assessments was much lower at 42%. This latter finding is consistent with that of Su et al. who reported that 42% of 26 patients with genetically confirmed HI/HA syndrome had abnormal scores on formal neuropsychological testing using the Chinese versions of the Gesell Developmental Schedules and Wechsler Intelligence Scale for Children [8]. MacMullen et al. similarly reported that 37% of 19 patients with HI/HA syndrome had documented abnormal neurodevelopment [7]. These findings contrast with those of Bahi-Buisson et al. [5]. In their retrospective chart review of 22 patients with HI/HA syndrome, 77% were reported to have intellectual disability, defined as an IQ score of 75 or lower, and 77% had developmental delays [5]. Differences in the reported rates of neurodevelopmental problems between studies may be attributable to the different assessment methods utilized. In addition, small sample sizes and patient heterogeneity likely contribute to imprecision in prevalence estimates.

In our study, neurocognitive outcomes were assessed by report and through self- or parent-completed validated measures, not through formal neurocognitive testing. Reported historical rates of developmental delays and learning disorders within our population did not correspond to abnormal results on the neurocognitive measures. This could have occurred because the neurocognitive measures administered would not detect resolved, historical developmental differences (ie: gross motor or speech delay). Other possible explanations for this finding include historical overreporting of deficits, response bias, and/or self or parent-overestimation of abilities on the neurocognitive measures. It is also possible that more circumscribed deficits (e.g., presence of specific learning disabilities or memory impairment) are prevalent in this population but not reflected in composite scores on neurocognitive rating scales used in this study. Formal, performance-based neuropsychological testing would address some of these limitations and help elucidate this further. Additionally, collection of ratings for a larger sample of individuals would allow for more detailed subscale analysis of domain-specific neurocognitive strengths and weaknesses.

Epilepsy in HI/HA syndrome is common, occurring, in 42–64% of those affected [5, 6, 8, 18, 19]. Atypical absence seizures in the absence of hypoglycemia were initially described by Raizen et al. in 2005 [6]. These seizures, which have electrographic features of generalized irregular spike and wave discharges at 3 to 6 Hz corresponding to eye blinks, eye rolling, or staring, have since increasingly been recognized in patients with HI/HA syndrome [5, 20–22]. While all subjects in this study reported early-onset seizures, determination of the prevalence of childhood epilepsy, versus recurrent hypoglycemic seizures, was limited by patient and parent recall. Using antiepileptic drug use as a proxy measure, the prevalence of childhood epilepsy in this study is consistent with prior reports.

Only one subject was diagnosed with absence epilepsy, and no subjects had electrographic seizures recorded on EEG. However, characteristic interictal EEG findings of generalized, irregular spike and wave discharges were observed in 50%, despite a reported history of seizure resolution in nearly all subjects. It is thus unclear whether the reported history of seizure resolution with age observed in this study reflects improvement in glycemic control with age and/or treatment, a variable natural history of epilepsy within HI/HA syndrome, or under-recognition of seizures. The high frequency of interictal EEG findings observed, combined with the subtle clinical manifestations of absence seizures raises particular concern for the latter of these and highlights the importance of longitudinal neurological assessment by specialists familiar with this disorder.

It has been proposed that the characteristic neurological features of the HI/HA syndrome result from abnormal CNS glutamate balance due to GDH overactivity [18, 20]. In a transgenic mouse model in which *GLUD1* was overexpressed in neurons, hippocampal glutamate levels measured by MRS were modestly increased in transgenic compared to wild-type mice [23]. A limitation in extrapolating these findings to humans with HI/HA syndrome is that GDH expression has been reported to be much greater in astrocytes than neurons [4]. In contrast, MRS findings from four related individuals with HI/HA syndrome were reported by Bahi Buisson, et al. in 2008, and all had normal glutamine peak (glutamate shows spectral overlap with glutamine, particularly at low field strength) [20].

While MRS has been the most commonly utilized method to evaluate CNS glutamate in vivo and has allowed for important insights into brain biochemistry, its utility in measuring glutamate is limited by both the low concentration of glutamate in the brain compared to water and spectral overlap with glutamine. The GluCEST technique has higher sensitivity and spatial resolution for measuring brain glutamate than MRS [24]. Using GluCEST MRI to explore the potential role of aberrant glutamate signaling in HI/HA syndrome, we found higher variability in both hippocampal GluCEST asymmetry and in peak hippocampal GluCEST values in HI/HA subjects compared to the healthy reference population. These findings provide evidence of a difference in distribution of hippocampal glutamate, as measured by GluCEST, in individuals with HI/HA syndrome as compared to unaffected individuals. Statistically significant differences in median hippocampal GluCEST AI and median peak hippocampal GluCEST were not observed, and additional data from a larger, future study is thus needed to explore how to best quantify differences in this population.

The observed differences in distribution of hippocampal GluCEST measures, along with the marked asymmetrical elevation in GluCEST observed in a subset of HI/HA subjects, suggests the possibility of subpopulations within HI/HA syndrome. A correlation between brain glutamate pattern and neurological phenotype did not emerge in this small study. A trend between abnormal hippocampal GluCEST signal and lower plasma ammonia levels was suggested. While intriguing given the enzymatic role of GDH in the interconversion of glutamate to alpha-ketoglutarate and ammonia, this statistically insignificant finding should be interpreted with particular caution, as it was partially driven by a single subject with normal plasma ammonia and mosaicism for an activating *GLUD1* mutation.

The degree of GluCEST signal asymmetry observed was an unexpected finding, particularly given the proposed pathophysiologic mechanism. Specifically, differential expression of GDH between the left and right hemispheres would not be expected. Although apparent hemispheric differences in hippocampal GluCEST signal could result from differences in imaging slice location in each hemisphere, this would neither fully explain the degree of elevation observed in the subset of subjects with lateralized high GluCEST signal, nor would it be expected to occur differentially among HI/HA subjects as compared to the reference population. Future work is needed to confirm and further explore these findings.

A genotype–phenotype association between mutations in exons 6 and 7 of the *GLUD1* gene and epilepsy was reported by Bahi-Buisson et al. in 2008 and Kapoor et al. in 2009 [5, 18]. Since then, however, this association has not been substantiated [8, 25]. Similarly, an apparent genotype–phenotype association did not emerge in our study with regard to neurocognitive outcomes, EEG, or GluCEST findings. Indeed, a substantial amount of phenotypic heterogeneity was observed, even within families.

There are several limitations to this study. The cross-sectional design, small sample size, and weight-based MRI restriction—which effectively excluded infants and young children—prohibited assessment of potential age and development-related differences. Neurodevelopmental and seizure history data were collected through subject and parent interview, which is subject to bias. Use of a single-slice imaging method for GluCEST MRI may have contributed to hemispheric differences in the measured GluCEST signal. Additionally, the single slice method precluded analysis of the entire hippocampus in addition to other brain structures potentially involved in the neuropathology of HI/HA syndrome. We evaluated hippocampal GluCEST because *GLUD1* is expressed in the hippocampus, which is a neural hub for learning and memory, and because hippocampal GluCEST data was available from a healthy reference population for comparison [10, 26–28]. Whole brain, volumetric GluCEST techniques are currently under development and could be used to address these limitations in the future. Since controls were not enrolled as part of this study, comparisons were made to the general population for neurocognitive assessment scores and to a historic healthy reference population for GluCEST outcomes. The use of historic neuroimaging controls could have introduced bias due to unmeasured temporal differences. Comparison to individuals with other hyperinsulinemic disorders could control for hypoglycemia-related effects and would likely prove more useful in evaluating the mechanisms underlying the unique neurologic phenotype of the HI/HA syndrome.

Larger studies including patients with other forms of HI, age-matched controls, and formal neuropsychological testing would help address these limitations and place this exploratory study’s findings into greater context. This type of work is currently ongoing.

## Conclusions

Our findings support the importance of longitudinal neuropsychological assessment for individuals with HI/HA syndrome by specialists familiar with this disorder. Additionally, these findings demonstrate the potential application of the GluCEST technique to investigate persistent knowledge gaps in the neuropathophysiological mechanisms underlying the unique phenotype of the HI/HA syndrome.

## Supplementary Information

**Additional file 1.** Hippocampal GluCEST values.

## Data Availability

Data that support the findings of this study are included in this article and its supplementary material file. Further enquiries can be directed to the corresponding author.

## References

[1] Snider KE, Becker S, Boyajian L, Shyng SL, MacMullen C, Hughes N (2013). Genotype and phenotype correlations in 417 children with congenital hyperinsulinism. J Clin Endocrinol Metab.

[2] Stanley CA, Lieu YK, Hsu BY, Burlina AB, Greenberg CR, Hopwood NJ (1998). Hyperinsulinism and hyperammonemia in infants with regulatory mutations of the glutamate dehydrogenase gene. N Engl J Med.

[3] Hudson RC, Daniel RM (1993). l-Glutamate dehydrogenases: distribution, properties and mechanism. Comp Biochem Physiol B.

[4] Spanaki C, Kotzamani D, Plaitakis A (2017). Widening spectrum of cellular and subcellular expression of human GLUD1 and GLUD2 glutamate dehydrogenases suggests novel functions. Neurochem Res.

[5] Bahi-Buisson N, Roze E, Dionisi C, Escande F, Valayannopoulos V, Feillet F (2008). Neurological aspects of hyperinsulinism–hyperammonaemia syndrome. Dev Med Child Neurol.

[6] Raizen DM, Brooks-Kayal A, Steinkrauss L, Tennekoon GI, Stanley CA, Kelly A (2005). Central nervous system hyperexcitability associated with glutamate dehydrogenase gain of function mutations. J Pediatr.

[7] MacMullen C, Fang J, Hsu BY, Kelly A, de Lonlay-Debeney P, Saudubray JM (2001). Hyperinsulinism/hyperammonemia syndrome in children with regulatory mutations in the inhibitory guanosine triphosphate-binding domain of glutamate dehydrogenase. J Clin Endocrinol Metab.

[8] Su C, Liang XJ, Li WJ, Wu D, Liu M, Cao BY (2018). Clinical and molecular spectrum of glutamate dehydrogenase gene defects in 26 Chinese congenital hyperinsulinemia patients. J Diabetes Res.

[9] Cai K, Singh A, Roalf DR, Nanga RP, Haris M, Hariharan H (2013). Mapping glutamate in subcortical brain structures using high-resolution GluCEST MRI. NMR Biomed.

[10] Davis KA, Nanga RP, Das S, Chen SH, Hadar PN, Pollard JR (2015). Glutamate imaging (GluCEST) lateralizes epileptic foci in nonlesional temporal lobe epilepsy. Sci Transl Med..

[11] Roalf DR, Nanga RPR, Rupert PE, Hariharan H, Quarmley M, Calkins ME (2017). Glutamate imaging (GluCEST) reveals lower brain GluCEST contrast in patients on the psychosis spectrum. Mol Psychiatry.

[12] Nanga RPR, DeBrosse C, Kumar D, Roalf D, McGeehan B, D'Aquilla K (2018). Reproducibility of 2D GluCEST in healthy human volunteers at 7 T. Magn Reson Med.

[13] Harrison P, Oakland T (2015). Adaptive behavior assessment system, third edition (ABAS-3).

[14] Achenbach TM (2009). The Achenbach system of empirically based assessment (ASEBA): development, findings, theory, and applications.

[15] Gioia GA, Isquith PK, Guy SC, Kenworthy L (2015). Behavior rating inventory of executive function-second edition (BRIEF2).

[16] Roth RM, Gioia GA (2005). Behavior rating inventory of executive function-adult version (BRIEF-A).

[17] Yushkevich PA, Piven J, Hazlett HC, Smith RG, Ho S, Gee JC (2006). User-guided 3D active contour segmentation of anatomical structures: significantly improved efficiency and reliability. Neuroimage.

[18] Kapoor RR, Flanagan SE, Fulton P, Chakrapani A, Chadefaux B, Ben-Omran T (2009). Hyperinsulinism–hyperammonaemia syndrome: novel mutations in the GLUD1 gene and genotype–phenotype correlations. Eur J Endocrinol.

[19] De Lonlay P, Benelli C, Fouque F, Ganguly A, Aral B, Dionisi-Vici C (2001). Hyperinsulinism and hyperammonemia syndrome: report of twelve unrelated patients. Pediatr Res.

[20] Bahi-Buisson N, El Sabbagh S, Soufflet C, Escande F, Boddaert N, Valayannopoulos V (2008). Myoclonic absence epilepsy with photosensitivity and a gain of function mutation in glutamate dehydrogenase. Seizure.

[21] Nakano K, Kobayashi K, Okano Y, Aso K, Ohtsuka Y (2012). Intractable absence seizures in hyperinsulinism–hyperammonemia syndrome. Pediatr Neurol.

[22] Perez Errazquin F, Sempere Fernandez J, Garcia Martin G, Chamorro Munoz MI, Romero AM (2011). Hyperinsulinism and hyperammonaemia syndrome and severe myoclonic epilepsy of infancy. Neurologia.

[23] Bao X, Pal R, Hascup KN, Wang Y, Wang WT, Xu W (2009). Transgenic expression of Glud1 (glutamate dehydrogenase 1) in neurons: in vivo model of enhanced glutamate release, altered synaptic plasticity, and selective neuronal vulnerability. J Neurosci.

[24] Cai K, Haris M, Singh A, Kogan F, Greenberg JH, Hariharan H (2012). Magnetic resonance imaging of glutamate. Nat Med.

[25] Ninkovic D, Sarnavka V, Basnec A, Cuk M, Ramadza DP, Fumic K (2016). Hyperinsulinism–hyperammonemia syndrome: a de novo mutation of the GLUD1 gene in twins and a review of the literature. J Pediatr Endocrinol Metab.

[26] Spanaki C, Kotzamani D, Petraki Z, Drakos E, Plaitakis A (2014). Heterogeneous cellular distribution of glutamate dehydrogenase in brain and in non-neural tissues. Neurochem Res.

[27] Human Protein Atlas. Available from http://www.proteinatlas.org.

[28] Uhlen M, Fagerberg L, Hallstrom BM, Lindskog C, Oksvold P, Mardinoglu A (2015). Proteomics. Tissue-based map of the human proteome. Science.

