# Correction: Characterizing the neurological phenotype of the hyperinsulinism hyperammonemia syndrome

**DOI:** 10.1186/s13023-022-02466-8

**Published:** 2022-08-18

**Authors:** Elizabeth Rosenfeld, Ravi Prakash Reddy Nanga, Alfredo Lucas, Andrew Y. Revell, Allison Thomas, Nina H. Thomas, David R. Roalf, Russell T. Shinohara, Ravinder Reddy, Kathryn A. Davis, Diva D. De León

**Affiliations:** 1 Division of Endocrinology and Diabetes, Children’s Hospital of Philadelphia, 3500 Civic Center Boulevard, Philadelphia, PA 19140 USA; 2 Congenital Hyperinsulinism Center, Children’s Hospital of Philadelphia, Philadelphia, PA USA; 3 Department of Pediatrics, Perelman School of Medicine, University of Pennsylvania, Philadelphia, PA USA; 4 Center for Advanced Metabolic Imaging in Precision Medicine, Hospital of the University of Pennsylvania, Philadelphia, PA USA; 5 Penn Center for Neuroengineering and Therapeutics, University of Pennsylvania, Philadelphia, PA USA; 6 Behavioral Neuroscience Core, Center for Human Phenomic Science, Children’s Hospital of Philadelphia, Philadelphia, PA USA; 7 Department of Child and Adolescent Psychiatry and Behavioral Sciences, Children’s Hospital of Philadelphia, Philadelphia, PA USA; 8 Department of Psychiatry, University of Pennsylvania Perelman School of Medicine, Philadelphia, PA USA; 9 Penn Statistics in Imaging and Visualization Center, Department of Biostatistics, Epidemiology, and Informatics, University of Pennsylvania Perelman School of Medicine, Philadelphia, PA USA; 10 Center for Biomedical Image Computing and Analytics, Department of Radiology, University of Pennsylvania Perelman School of Medicine, Philadelphia, PA USA; 11 Department of Neurology, University of Pennsylvania Perelman School of Medicine, Philadelphia, PA USA

## Correction to: Orphanet Journal of Rare Diseases (2022) 17:248 10.1186/s13023-022-02398-3

Following publication of the original article, we have been notified that there is an error in one of the author’s first names and ORCID should be added to one of the authors. It should be as per below: First name is Russell, middle initial is T., and family name is Shinohara.Diva D. De León should have ORCID mentioned - 0000-0003-1225-8087.

